# When Grammar and Parsing Agree

**DOI:** 10.3389/fpsyg.2018.00336

**Published:** 2018-04-03

**Authors:** Simona Mancini

**Affiliations:** Basque Center on Cognition Brain and Language, San Sebastián, Spain

**Keywords:** agreement, online/offline processing, misalignment, ERPs, eye tracking

## Introduction

Theoretical and experimental research on language has nowadays reached an extraordinary level of complexity. Generative linguistics has produced detailed maps of syntactic structures and computations that have significantly contributed to our knowledge of language architecture as an abstract system (Rizzi, 2012; Everaert et al., 2015). Psycho-/neurolinguistics has developed complex experimental designs and techniques that have made it possible to study linguistic processing with millisecond and voxel precision (Friederici, 2002, 2011; Hagoort, 2005; Bornkessel-Schlesewsky and Schlesewsky, 2009, 2013). Unfortunately, the dialogue between the two disciplines has not always been constant, with theoretical linguistics often proceeding without drawing on experimental results, and psycho-/neurolinguistics limitedly relying on linguistic theory (see discussion in Ferreira, 2005; Poeppel and Embick, 2005; Jackendoff, 2007; Embick and Poeppel, 2015). This has resulted in a sharp separation between the formal/computational level of linguistic analysis and the functional/neuro-anatomical investigation of language, limiting the depth with which we can investigate what we know and what we do with language.

There have been proposals for the development of research programs in which linguistics and psycho-/neurolinguistics engage in a tighter dialog, with the aim of highlighting the relation between theoretical formalizations, cognitive mechanisms and their neurobiological implementation (Marr, 1982). The current opinion article shows how such a research program can be successfully implemented.

## Grammar and parsing: convergence or misalignment?

The domain where the distance between generative linguistics and psycho-/neurolinguistics is more clearly manifest is the definition of the role of grammar and its relation with parsing. This divide is reflected in the opposition between the “*two-systems view*” and the “*one-system view*” (Lewis and Phillips, 2015). Under the former account, the grammar represents a static body of knowledge whose content is reflected in speakers' ability to verify offline the acceptability of a sentence, without the memory and executive function limitations to which parsing is typically subject (Chomsky, 1965). Online parsing represents a separate system that operates on a different set of computations, such as pseudo-grammars based on heuristics or good-enough representations (Townsend and Bever, 2001; Ferreira and Patson, 2007). Reliance on distinct sets of computations/representations explains why the sentence processing literature frequently reports misalignments between online and offline responses. These are cases of sentences that are typically regarded as ungrammatical in offline judgments, but that are processed as if they were grammatical in online measures, as happens in garden-path or agreement attraction processing. The alternative perspective - the one-system view - claims that grammar and parsing are part of a unitary cognitive system, with the former being recruited during the different stages of real-time processing to build the representations that comprehension and production produce (Embick and Poeppel, 2015; Lewis and Phillips, 2015). A straightforward argument for a unitary view of grammar and parsing is represented by the convergence between online and offline responses to grammatical anomalies (see Sprouse and Almeida, 2013 for a review; Lewis and Phillips, 2015), but also by the extreme sensitivity to incremental structure building operations that brain oscillations and neural substrate activation have (Pallier et al., 2011; Ding et al., 2016; Nelson et al., 2017).

Given the sensitivity with which structural information is tracked during online processing, it is therefore surprising that online and offline responses do not always align. One possibility, and the one that is explored here, is that online and offline responses simply represent distinct snapshots of a process that unfolds in time and that goes through different computational stages, rather than resulting from a separate system (Phillips and Lewis, 2013; Lewis and Phillips, 2015, p. 30). If this is so, misalignment is then compatible with the one-system view. The current opinion article seeks to further explore the relation between grammar and parsing by looking inside the stages of agreement computation as revealed by techniques with exquisite temporal resolution such as event-related potentials (ERPs) and eye-tracking.

## Convergence and misalignment in agreement

Agreement represents a paradigmatic case for the study of the relation between grammar knowledge and online processing. As will be shown, across the different experimental paradigms available, online and offline data both align and misalign.

In its standard configuration, agreement manifests itself as feature covariance between, for example, a nominal and a verbal element, as shown in (1) below for Spanish.

(1) Los lingüistas_3.pl_ *escriben*_3.pl_ artículos interesantes.
     The linguists write very interesting articles

For agreement to be established, the grammar minimally requires the identification of a nominal element with subject properties and matching features. A feature checking operation then verifies the consistency of nominal and verbal morphosyntactic information.

Regardless of the task, anomalies such as the person disagreement in (2) for Spanish are rapidly and unmistakably detected by speakers, in line with the one-system view.

(2) ^*^El lingüista_3.sg_ escribes_2.sg_ artículos interesantes
     ^*^The linguist write interesting articles

Crosslinguistically, self-paced reading and eye-movement measures reveal increased reading times on the anomalous word (Deutsch and Bentin, 2001; Braze et al., 2002; De Vincenzi et al., 2003; Mancini et al., 2014a). ERPs show the emergence of a late positive effect, the P600. Underscoring its domain-generality, the P600 has been recently interpreted as an index of repair/reanalysis processes after a conflict has been detected between the expected and the perceived input (Van de Meerendonk et al., 2009). Person anomalies of the type in (1) also engender broad negative effects with a centro-posterior maximum, in line with the distribution of N400 effects (but see Silva-Pereyra and Carreiras, 2007 for anterior negative effects; Zawiszewski and Friederici, 2009; Mancini et al., 2011a,b; Zawiszewski et al., 2016)^1^^.^ Given the semantic nature of N400 effects (see Kutas and Federmeier, 2011 for a review), these effects have been taken to reflect the detection of an anomaly that extends beyond the morphosyntactic domain, to involve the assignment of fundamental discourse roles (e.g., speaker and addressee) required for the interpretation of person agreement (Sigurdsson, 2004; Bianchi, 2006; Mancini et al., 2011a).

Yet, the literature on agreement also describes cases of subject-verb agreement processing that are allegedly problematic for the one-system view, such as (4). Crucially, their availability allows us to enrich our understanding of the grammar-parsing relation. Let us see how.

Patterns like (4) are known as unagreement (Hurtado, 1985)^2^ Despite the subject-verb person mismatch, this sentence is nevertheless grammatical. Grammaticality is ensured by superimposing the 1st/2nd person plural interpretation onto the 3rd person subject.

(3) Los lingüistas_3.pl_ escribimos_1.pl_/escribís_2.pl_ artículos interesantes
     We/You linguists write interesting articles

Unagreement processing has been investigated with several experimental techniques –among which online and offline grammaticality judgments, ERPs and eye-tracking- with which it has been compared to standard agreement and erroneous agreement such as (1) and (2) above^3^. The unagreement paradigm presents a straightforward advantage compared to anomaly-detection paradigms. Unlike outright anomalies, unagreement grammatical mismatch makes it possible to functionally and temporally identify when feature consistency is checked from when the overall 1st/2nd person interpretation is assigned. This is of fundamental relevance, if we want to assess whether timed grammaticality judgments capture internal stages of computations.

Although the available theoretical analyses of unagreeement each offer a different explanation of what licenses unagreement feature mismatch (see review in Ackema and Neeleman, 2013), all converge in treating this pattern as an instance of regular agreement. From the perspective of grammar, no apparent subject-verb disagreement is therefore involved. Nevertheless, online and offline grammaticality judgments on unagreement do misalign. Speakers have been found to rate unagreement as equally grammatical as standard agreement when no time pressure is imposed on the experimental task, while a significant drop in accuracy is evidenced during a speeded evaluation (Mancini et al., 2014b, Experiment 2).

To find out where this misalignment stems from, unagreement processing should be closely tracked, in order to identify possible temporally and functionally distinct stages. The exquisite temporal resolution of ERPs and eye-tracking can help us separate in time the cognitive process that leads to unagreement comprehension. If we can demonstrate that the effect of unagreement featural mismatch changes across processing stages, we can safely conclude that misalignment arises from intermediate computational stages. More precisely, we should observe an early stage during which unagreement causes significant processing penalties compared to standard agreement, with early negative effects in ERPs and increased first-pass reading times in eye tracking. This should be followed by a stage in which unagreement grammaticality is acknowledged, and no disruption is observed relative to standard agreement (neither second-pass reading effects nor late positive ERP effects).

Alternatively, if online and offline responses result from different systems, constant misalignment could emerge across computational stages. Eye-tracking and ERP correlates of unagreement could closely pattern with person violation, showing first- and second-pass reading effects, and biphasic early negativity-late positivity ERP patterns.

Behavioral and electro-physiological investigation has identified two temporally and functionally distinct stages for unagreement processing, during which the response to these grammatical mismatches vary, in line with the first hypothesis. At an early stage, reading times on the verb increase (Mancini et al., 2014b), and early negativities in ERPs (Mancini et al., 2011b) arise. The absence of late positivities and of second-pass effects for unagreement (relative to standard agreement, Mancini et al., 2011b, 2014b), Experiment (4) clearly indicates a degree of grammaticality. Overall, a mismatch is detected at one moment, but not a few hundred milliseconds after, when the overall 1st/2nd person plural interpretation of the relation is derived. Can online grammatical evaluations represent the snapshot of an incomplete computation? The answer is, in my opinion, affirmative: any deviation of the online from the offline evaluation of unagreement is circumscribed within a temporary stage, arguably corresponding to when feature checking operations are implemented. Importantly, unlike person violations, unagreement mismatch is not strong enough to alert conflict-monitoring process and trigger repair operations. This suggests that the parser is aware that the mismatch will be solved in a subsequent computational stage.

Misalignments in other well-studied phenomena of agreement processing—attraction for example—are not directly amenable to internal stages of computations (but see Franck et al., 2006; Franck, 2011). The illusion of grammaticality that is generated by the features of a structurally irrelevant noun (e.g., *cabinets* in “^*^The keys to the cabinets are rusty,” see Clifton et al., 1999; Pearlmutter et al., 1999; Franck et al., 2010, 2015; Dillon et al., 2013; Tanner et al., 2014, among others) has been recently attributed to the noisy architecture of the memory access system. Under this account, all available nominal objects are simultaneously probed for their match with verbal cues, and this can occasionally lead to the retrieval of the wrong subject (Wagers et al., 2009)^4^. Critically, the heterogeneity of factors that can predict misalignment of online/offline responses should not obscure the fact that in both unagreement and agreement attraction, real-time processing operates with the same computations required by the grammar in offline judgments, namely by identifying an element with subject properties and checking the consistency of its features against the verb.

## Conclusion

To conclude, this discussion on (un-)agreement processing shows that mere acknowledgment of mis-alignment between online and offline judgments is not sufficient to define the relation between what we know and what we do with language. Closer and deeper scrutiny of online processing has proved essential to connect theory, cognition and neurobiology of language, testifying how the interaction between these three levels of analysis can significantly contribute to advancing our knowledge of the architecture of language.

## Author contributions

The author confirms being the sole contributor of this work and approved it for publication.

### Conflict of interest statement

The author declares that the research was conducted in the absence of any commercial or financial relationships that could be construed as a potential conflict of interest.

## Footnotes

^1^Number violations generally elicit LAN effects (De Vincenzi et al., 2003; Barber and Carreiras, 2005; Rossi et al., 2005; Silva-Pereyra and Carreiras, 2007; Mancini et al., 2011a, among others; see Molinaro et al., 2011 for an extensive review).

^2^Unagreement is available in several Romance and non-Romance languages. An overview can be found in Corbett (2006) and Höhn (2016).

^3^Experimental investigation on unagreement has been so far conducted only in Spanish.

^4^Agreement attraction is a rather prolific subfield in the sentence comprehension (and production) literature. An exhaustive review of extant accounts is provided by Bock and Middleton (2011).
